# Online‐based software for guiding immediate implantation to replace a tooth with root resorption in the esthetic zone

**DOI:** 10.1002/ccr3.3117

**Published:** 2020-07-19

**Authors:** Krasimir Ivanov Chapanov, Georgi Veselinov Iliev, Stoyan Torezov Kazakov

**Affiliations:** ^1^ Department of Oral and Maxillofacial Surgery Faculty of Dental Medicine Medical University Sofia Bulgaria; ^2^ Department of Prosthetic Dental Medicine Faculty of Dental Medicine Medical University Sofia Bulgaria

**Keywords:** computer‐aided design, digital dentistry, implantology, prosthodontics

## Abstract

This case report describes an alternative approach for the planning of immediate implant placement and provisionalization, which uses a new online dental software that predicts the esthetic outcome.

## Graphical abstract

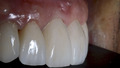

## 1 INTRODUCTION

The use of dental implants for single‐tooth replacement has shown high long‐term success rates.^1^ The choice of immediate or delayed implant placement may be made based on the esthetic expectations of the patient and the site of implant rehabilitation. The ideal indications for immediate implant placement include generally healthy patients with a low surgical risk and good oral hygiene, as well as the replacement of a single tooth in the nonesthetic zone.^2^ Immediate implant placement for single‐tooth replacement following extraction in the esthetic zone, however, is not a frequently used approach.

Under ideal conditions, immediate implant placement should use a flapless approach. Compared with the open‐flap approach, flapless implant surgery is associated with less hard‐ and soft‐tissue alterations.^3^ The implant should be placed in the correct three‐dimensional (3D) position, leaving at least 2 mm of space for the insertion of bone substitute between the implant surface and facial wall, as recommended by a recent International Team for Implantology (ITI) consensus statement.^4^ A gap of this dimension also facilitates the formation of new woven bone. This was demonstrated in a preclinical study, in which a wider void between the implant and the socket wall was associated with less marginal bone loss compared with a narrower void.^5^

Postextraction sites usually require the addition of bone substitute in order to improve the contour of the alveolar ridge. Current guidelines recommend the use of bone substitutes with a low resorption rate, such as deproteinized bone material, for filling the voids between the implant surface and surrounding alveolar bone.^6^

Advantages of immediate implant placement include a reduction in the number of required surgeries, lower morbidity, and shorter healing periods, all of which increase the attractiveness of this type of implant therapy to patients. Early implant placement, on the other hand, is performed 4‐8 weeks following tooth extraction and is associated with the following advantages: completion of soft‐tissue healing at the site of tooth extraction; completion of osteoclast activity; and spontaneous soft‐tissue thickening. Indications for early implant placement include the presence of a thin bone wall phenotype and thin gingival phenotype, as well as the absence of infection.^7^

Root resorption is a pathological process characterized by a progressive loss of dentine and cementum due to osteoclastic activity^8^ and can be classified as either internal or external. Both types are known to be initiated and maintained by a wide range of factors, including pulpal necrosis, trauma, periodontal treatment, excessive orthodontic treatment, tooth whitening agents, and chronic odontogenic infection.^9^ Regardless of the initial cause, the process is inflammatory in origin, and successful management depends on a thorough understanding of the diagnosis.

External resorption is often an incidental finding which is self‐limiting and can be simply monitored. It can be further classified as either external surface resorption, external inflammatory resorption, external replacement resorption, external cervical resorption or transient apical breakdown. While these pathological inflammatory processes can usually be successfully managed with conventional endodontic treatment, some cases may require surgical exposure and direct restoration of the superficial lesion.

Internal inflammatory resorption requires endodontic treatment, with special attention to ensure that an optimal root canal preparation and adequate root filling are performed.^10^ Nevertheless, in some cases, the inflammatory process proceeds and extraction of the tooth is inevitable. Immediate implant placement following tooth extraction may be considered the treatment of choice in such cases.^11^

The most creative part of the digital workflow process is the smile design. Meeting a patient's expectations in terms of esthetics presents as a major challenge for the dentist.^12^ Computer‐aided design and computer‐aided manufacturing (CAD/CAM) technology not only facilitates the process of milling custom abutments with different materials, but also the creation of a prototype of the patient's smile. This guides the treatment planning process and also aids discussion with the patient. Smile design software like Rebel Simplicity (Visagismlile Ltd.) creates a completely automated 3D digital mock‐up of the treatment outcome on STL files, which are used to print a resin model. A mock‐up for clinical try‐in can be fabricated with a silicon index and bis‐acryl resin, and this provides both a preview of the treatment outcome for the patient, as well as a guide for the clinician during the surgical placement of the implants.

The purpose of this case report is to describe a faster and easier approach for planning surgical and prosthetic treatment by using a new online‐based digital software that facilitates communication with patients and predicts the esthetic outcome.

## 2 CASE PRESENTATION

**Figure 1:**
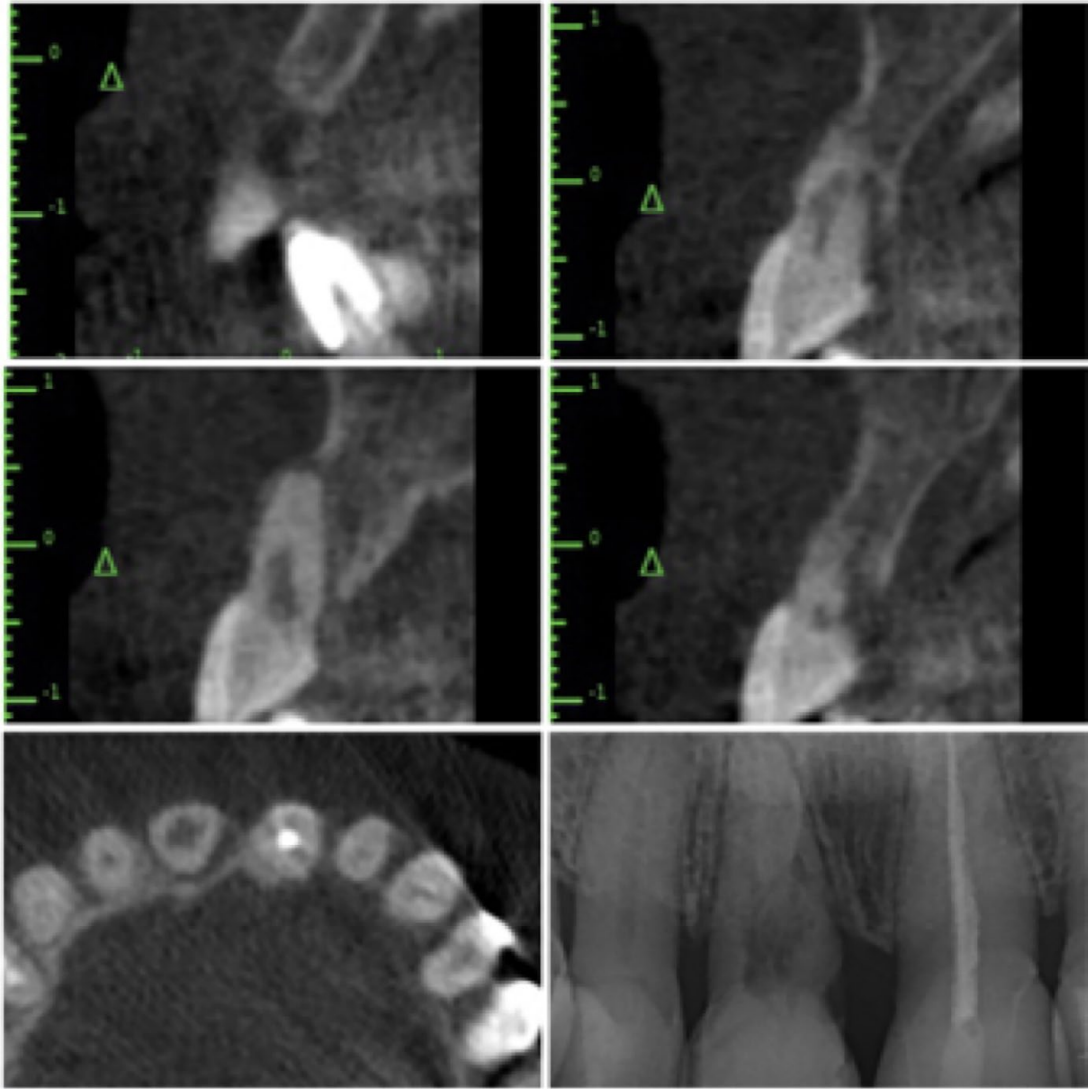
A diagnosis of internal and external inflammatory root resorption was made following cone‐beam computed tomography, periapical radiography, and pulp vitality testing

A 48‐year‐old woman presented with esthetic concerns regarding her smile, which were attributed to a high smile line, slight chipping of her porcelain crowns. The patient had noticed these problems for approximately 1 year. Tooth mobility was confirmed upon intraoral examination, and a probing depth of 9 mm was recorded at the mesiopalatal side of tooth 11. Additional investigations were subsequently performed. A small vertical bone defect was observed at the mesiopalatal side of tooth 11 on a cone‐beam computed tomography scan and a periapical radiograph. Areas of cementum and dentin resorption were found, in addition to adjacent bone loss. The pulp chamber was also enlarged at the cervical region. Tooth 11 was nonresponsive to pulp vitality testing (Figure 1).

After evaluation of the local factors and considering the fact that the clinical symptoms had been evident for more than 1 year, a treatment plan was proposed for the flapless extraction of tooth 11, followed by immediate implant placement with instant provisionalization.

**Figure 2:**
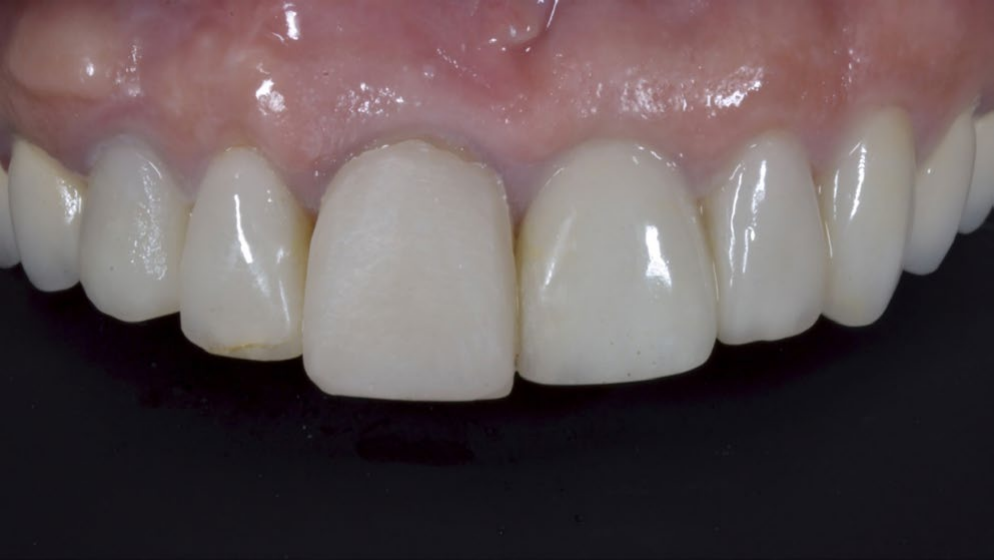
Treatment of every esthetic case should start by defining the incisal edge position of the maxillary central incisors. A composite mock‐up on one (or two) of the central incisors identifies the incisal edge position (occlusogingivally) and the position of the facial surface (buccolingually). This simple mock‐up was 3D digitally scanned together with the full maxillary arch

Full‐face photography was performed before and after implant placement. Digital scanning was conducted, and a single mock‐up defining the new incisal edge position was made for tooth 11. All data were transferred to an online‐based digital laboratory^12^ (Figure 2).

**Figure 3:**
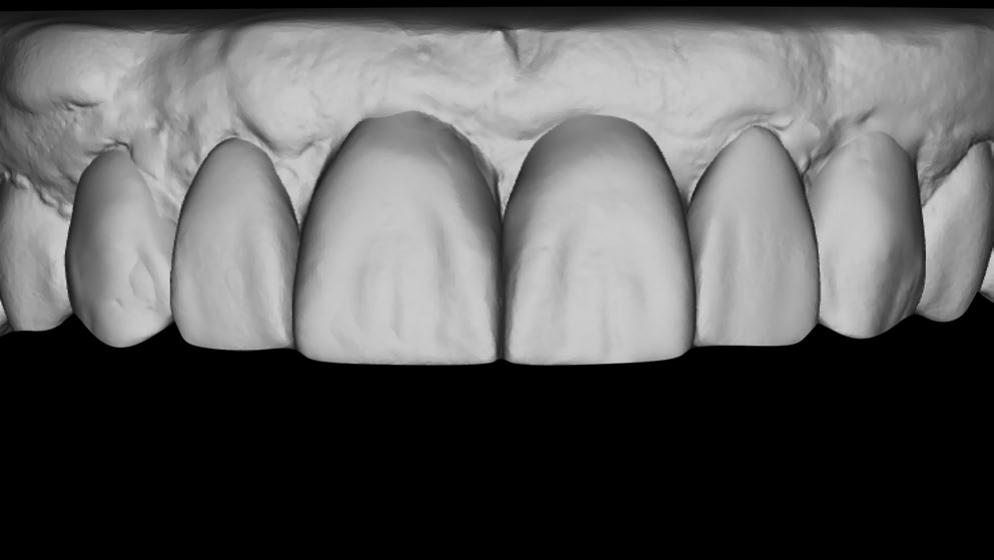
Smile design software can provide immediate recalculations and recreations of personalized 3D models of the teeth by morphing the individual tooth shapes from its 3D library within 15 min, through a complete hands‐free digital workflow. Every model is generated according to the proposed 2D configuration of the teeth. Users can visualize the 3D model in their browsers and can also download the models for use in a dental laboratory

We used an online‐based smile design software (Rebel Simplicity; Visagismlile Ltd.) to create 2D digital designs of the restorations, which reflected the patient's esthetic preferences and expectations. The 2D designs were used to guide the complete rendering of the 3D digital model for subsequent printing. Once the positions of the incisal edges of the incisors were defined, we created new 3D digital designs for the teeth proportions and extended the length of the teeth in the coronal and apical directions (Figure 3). The single mock‐up for the incisors was used as a reference for the automatic creation of a complete 3D digital mock‐up (on STL files) for the subsequent printing of a resin model.

**Figure 4:**
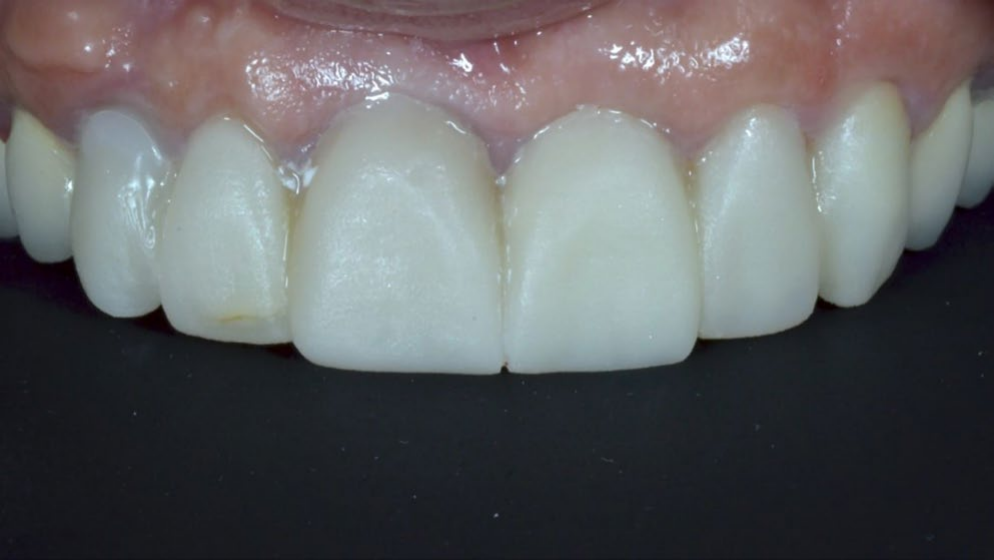
After the STL file is received in the digital laboratory, it can be 3D printed. When the 3D‐printed model is created, the dentist can easily transfer this design to the patient's mouth by making a silicone impression of the digital wax‐up and using a provisional material of choice

**Figure 5:**
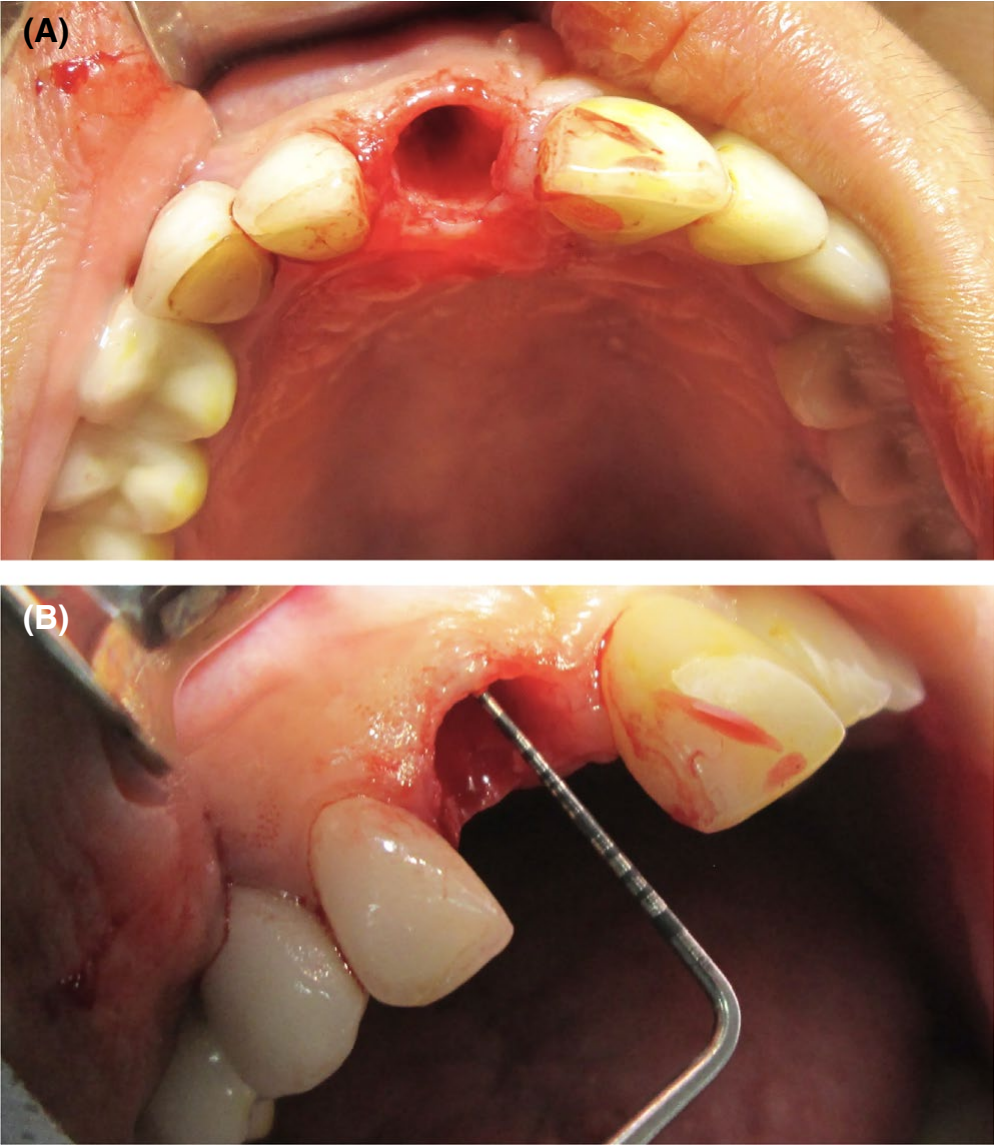
A and B, Tooth 11 was extracted under local anesthesia, with no complications. As the vestibular cortical bone was fenestrated, a full mucoperiosteal flap, with an incision between 11 and 12, was elevated

**Figure 6:**
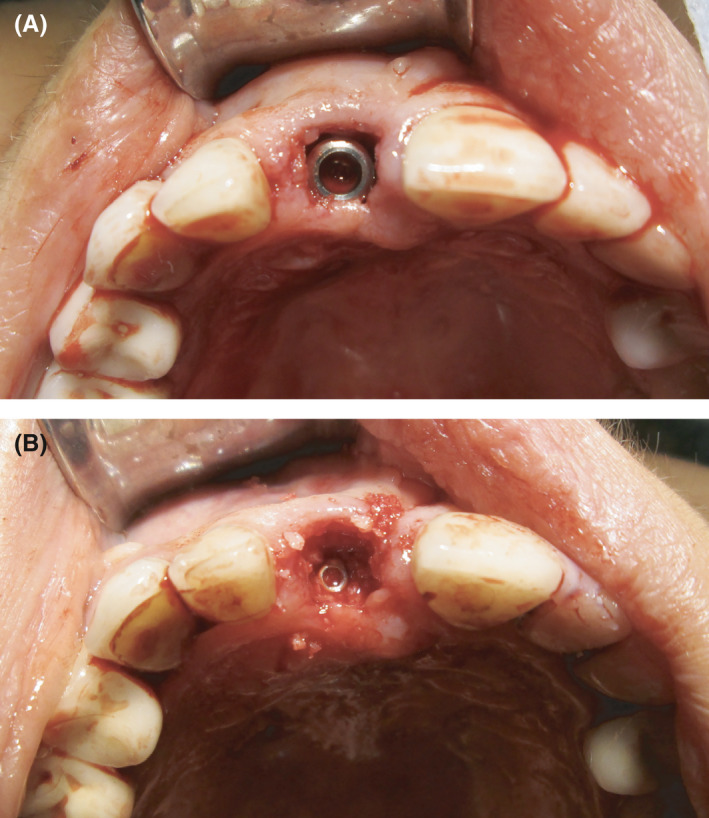
A and B, After implant placement, a xenograft material was applied between the flap and the implant in order to prevent bone loss during the healing period. A collagen membrane was placed to promote wound healing and regeneration

**Figure 7:**
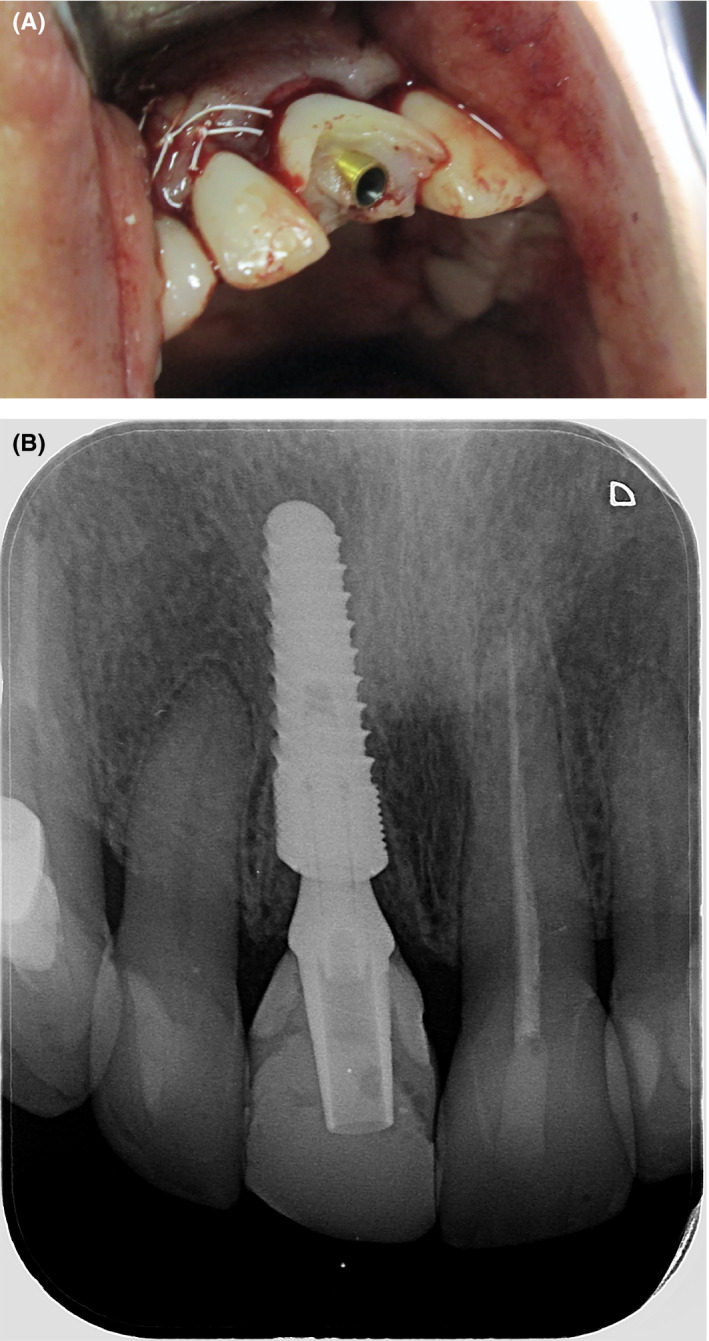
A and B, The flap was adapted and sutured with Teflon threads. A prefabricated temporary crown was cemented on the titanium abutment

**Figure 8:**
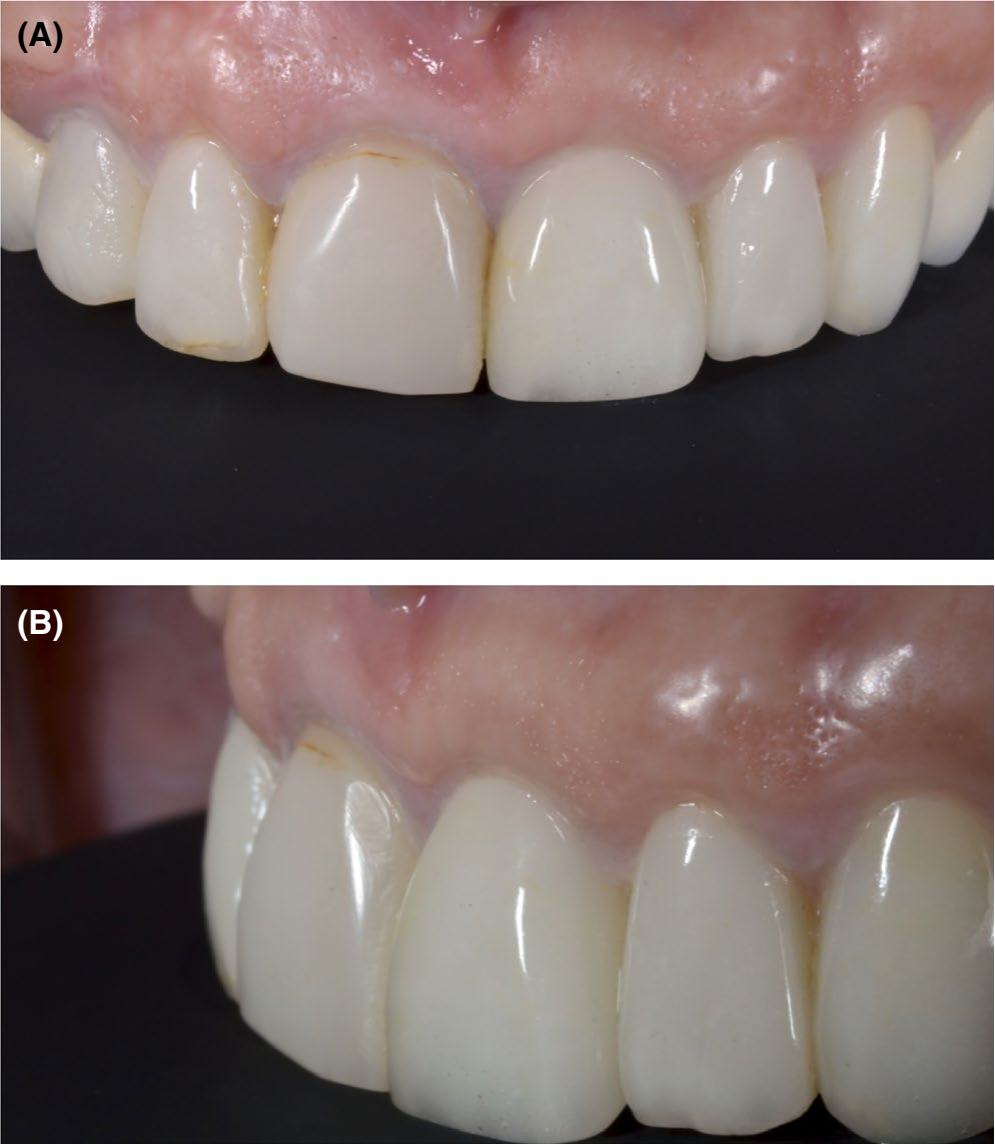
A and B, The result 1 y after immediate implant placement. The tissue volume was preserved, and the gingival contour was slightly higher than the adjacent tooth. This was easily corrected with the final restorations

A silicon index was made from the printed model, and a new mock‐up for clinical try‐in was fabricated with bis‐acryl resin (Figure 4). After the patient's approval of the mock‐up, preoperative antibiotics were administered (2 hours prior to the surgery; Augmentin 1000 mg), and tooth 11 was extracted under local anesthesia. As the vestibular cortical bone was observed to be thin and fenestrated during the implant procedure, a full mucoperiosteal flap was made with one vertical‐releasing incision between the right central and lateral incisors. A titanium dental implant (grade 4) with a diameter of 4.0 mm and a length of 10 mm was inserted with a torque of more than 45 Ncm to achieve a high primary stability (Naturall+; NICP 40 100; Euroteknika). A xenograft material (Gen‐Os; Osteobiol^®^) was applied to fill the space between the implant surface and the facial wall, in order to prevent bone alteration during the healing process. A collagen membrane (Evolution, Osteobiol^®^) was applied to promote wound healing and regeneration (Figures 5, 6, 7, 8), and the flap was adapted and sutured. Postoperative antibiotics (1000 mg Augmentin, GSK, twice daily for 7 days), as well as a mouth rinse (0.1% chlorhexidine, Eludril Classic, twice daily for 14 days), were prescribed.

Due to the sufficient primary stability of the implant, a provisional implant‐retained restoration was placed without immediate loading. The transitional zone of the crown was designed with a narrow and concave profile on the facial aspect and a slightly convex profile on the proximal surfaces to allow a greater volume of gingival tissue in the interproximal areas for support. Contact points with the adjacent teeth were tight and positioned 5‐6 mm above the bone level.

**Figure 9:**
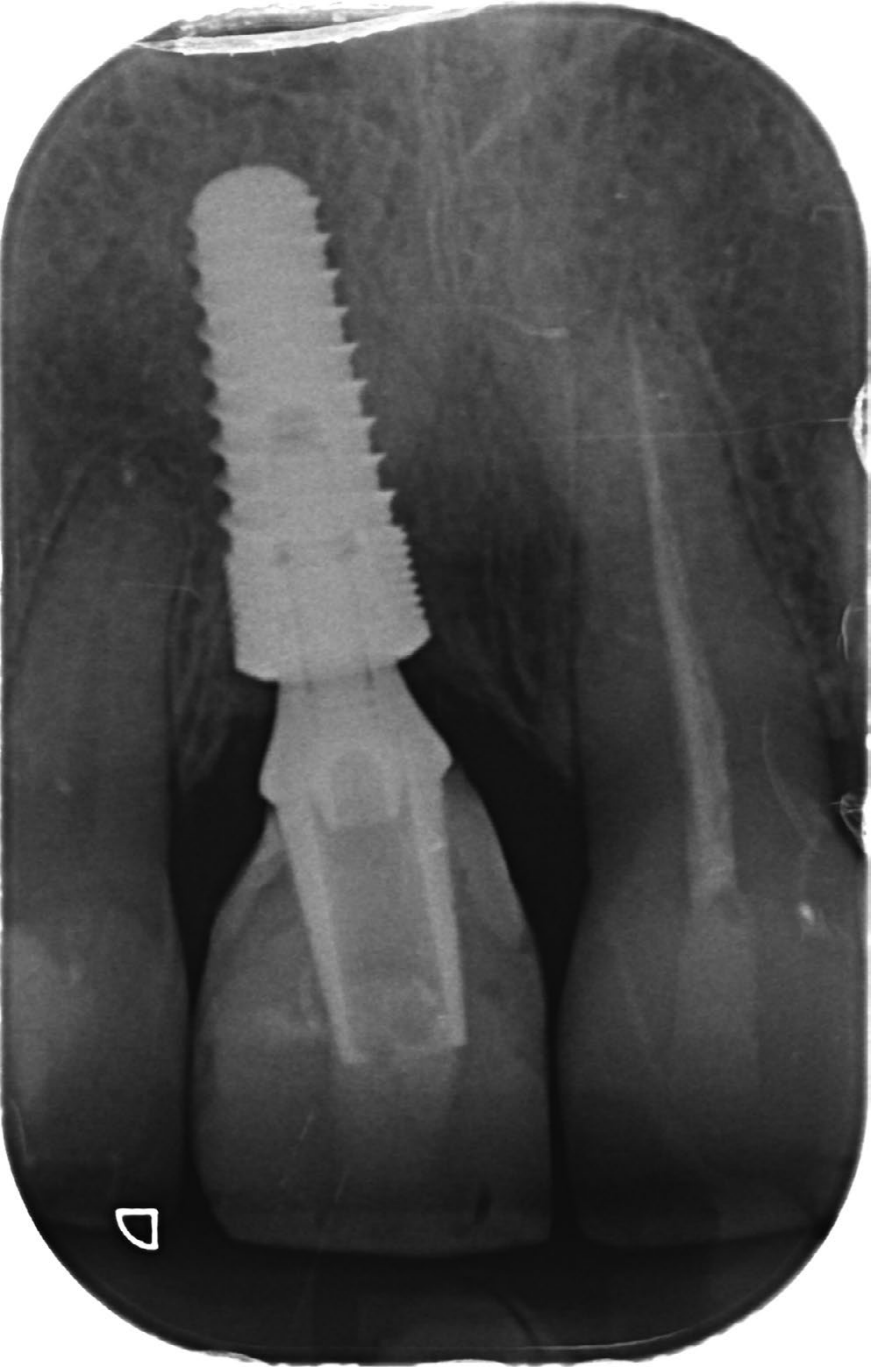
No bone loss in the zone was observed 1 y after implant placement

**Figure 10:**
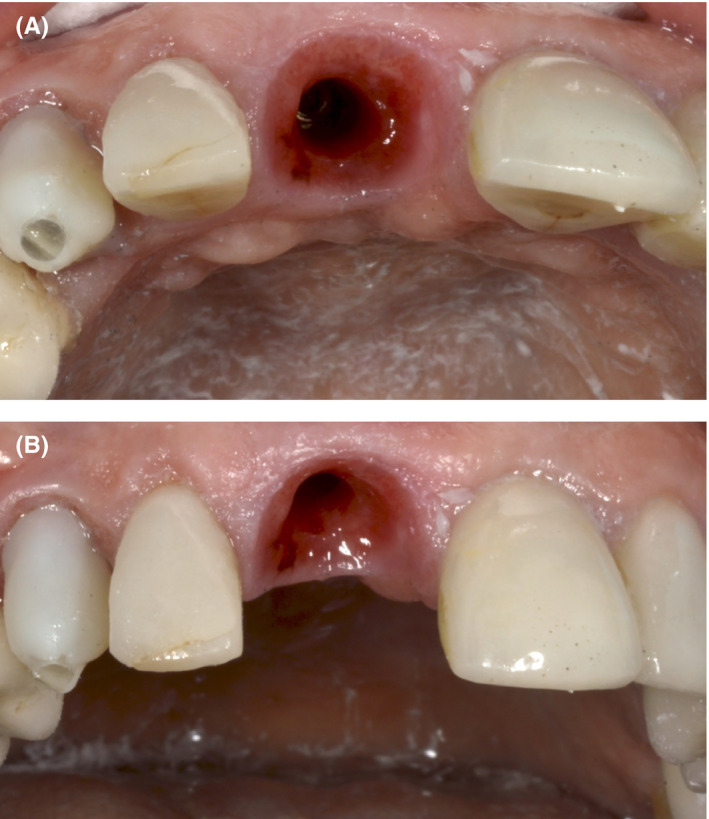
A and B, The tissue volume was preserved on the buccal aspect of the implant. The gingivae were healthy and had well‐formed contours

**Figure 11:**
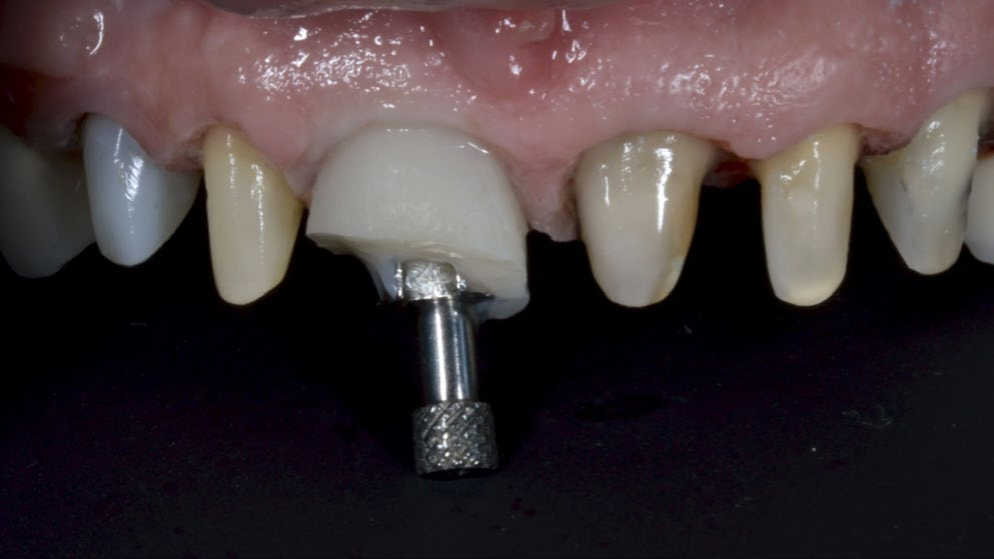
The contours of the gingival aspect of the provisional crown (on the implant abutment) provided the same degree of gingival tissue support, as that achieved during the maturation period

**Figure 12:**
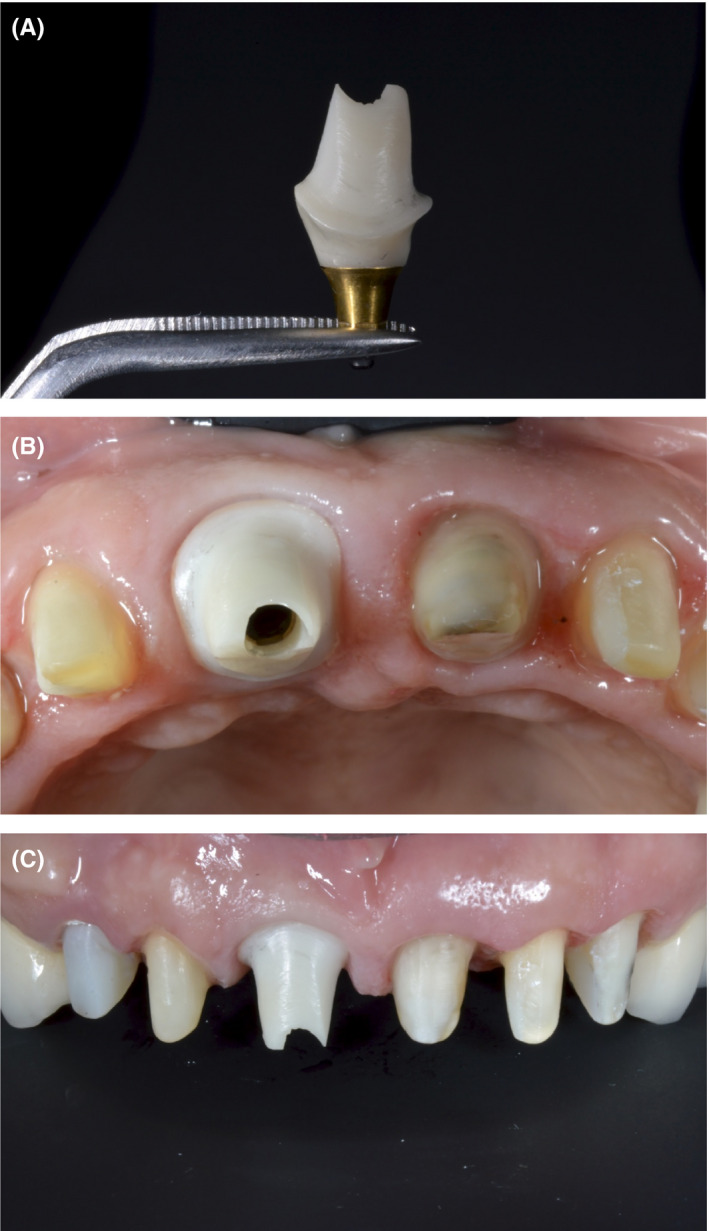
A‐C, The final indirect mock‐up (which was fully digitally designed as a 3D wax‐up) was tried in the patient's mouth. It was subsequently used to make an individualized zirconium abutment which was cemented onto the Ti‐base

At the 1‐year follow‐up, no soft‐tissue inflammation or marginal bone loss was observed (Figures 9 and 10). All anterior teeth (from canine to canine) were prepared for lithium disilicate crowns, and a final impression (that captured the transitional zone at the implant site) was taken (Figure 11). Upper and lower models were mounted in a semi‐adjustable articulator, and the distance between the zenith points of the anterior teeth was measured to determine the clinical gingival contour. The design of the tooth preparations and zirconium abutments was guided by the digital mock‐up (Figure 12). The individual restorations for the prepared teeth and implant abutment were subsequently designed, and a wax model was milled from the CAD data. Final restorations were fabricated and tried in. All crowns were glazed following chair‐side adjustments and cemented with a resin luting agent (RelyX™ Lutin Cement; 3M). We utilized a lithium disilicate crown for the zirconium abutment, as opposed to a screw‐retained monolithic zirconium crown, in order to more closely match the optical characteristics of the adjacent restorations.

## 3 DISCUSSION

Dental implants are a viable option following the extraction of teeth with a poor endodontic prognosis due to internal or external root resorption. This case report demonstrates successful outcomes for single‐tooth replacement in the esthetic zone, with immediate implantation and instant provisionalization, using a computer‐aided design and 3D printing approach. The use of smile design software facilitates not only surgical and prosthetic treatment planning, but also effective communication with patients, and helps to ensure that the final restoration meets the patients’ expectations.

The major determinants of treatment outcomes in the esthetic zone are the dimensions of the alveolar bone, as significant resorption occurs during the first 8‐12 weeks after tooth extraction.^13^, ^14^ Assessment of alveolar bone dimensions informs the optimal time for surgical implant placement.

Immediate implant placement requires special attention to ensure correct positioning, with the goal of achieving an optimal esthetic outcome. If ideal conditions are present, a flapless surgical approach is recommended.^15^ While this approach is associated with a significant risk of mucosal recession,^16^ the implant survival rate has been reported to be similar to that of the delayed approach.^17^

Early implant placement is performed 4‐8 weeks after tooth extraction. Soft‐tissue healing is completed between 4 and 8 weeks, depending on the amount of change in bone dimensions after tooth extraction. An advantage of this approach is the lack of any alterations of the alveolar crest width in the interproximal area. Furthermore, there is an increased thickness and keratinization of the gingival tissues at the future implant site; these factors facilitate subsequent bone augmentation and are associated with a lower risk of mucosal recession. However, a disadvantage of this approach is the increased number of clinical procedures required. Nevertheless, in our current practice, early implant placement is the most commonly used approach for tooth replacement in the esthetic zone, especially when patient expectations are high.

**Figure 13:**
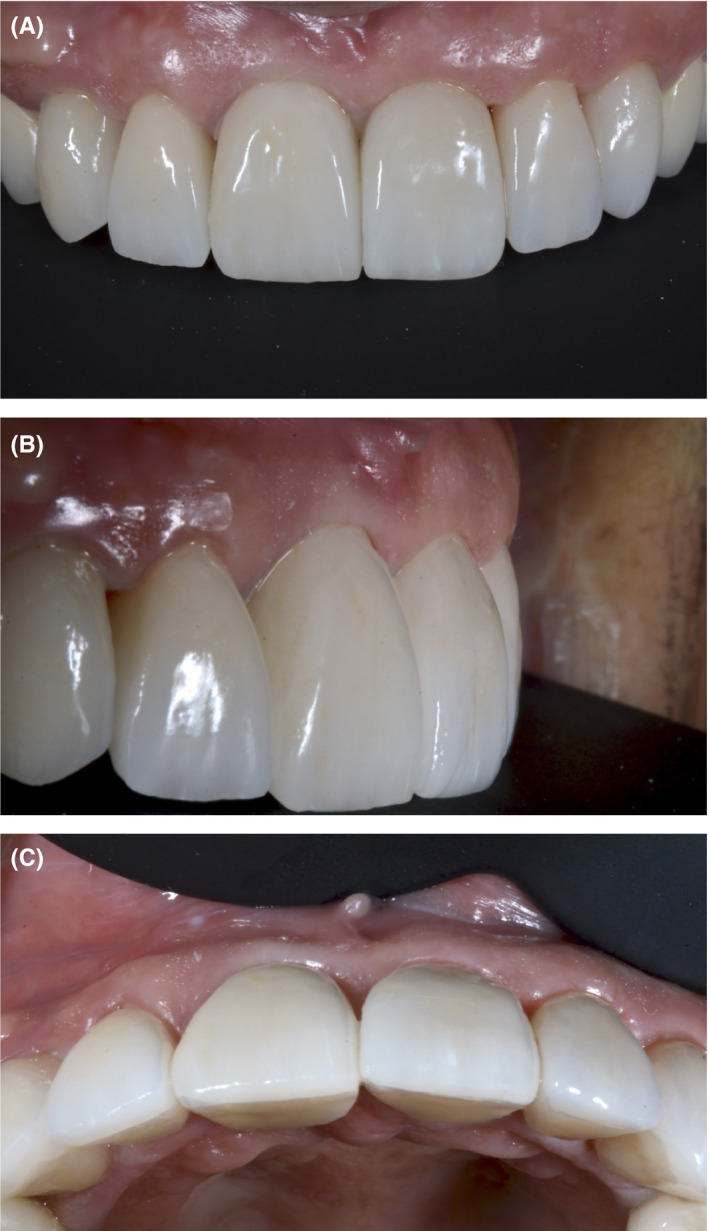
A‐C, The final result, showing the e.max porcelain crowns in place. The patient reported that the esthetic appearance of the prostheses approximated that of her natural teeth, and that she was extremely satisfied with her smile

Smile design software, such as Rebel Simplicity, can be used to resolve a number of dilemmas regarding the choice of surgical approach, as well as materials, positioning, and dimensions used for the prosthetic teeth. These choices are all taken into consideration with the aim of predicting and achieving the best possible functional and esthetic outcome. Using smile design software can lead to optimal clinical results, as it reduces the number of required chair‐side adjustments and patient complaints following treatment completion (Figure 13). A recent literature review conducted by Cervino et al^18^ highlighted the use of digital smile design software in different dental disciplines. They concluded that this technology has revolutionized traditional dental treatment, in areas ranging from treatment planning to the accurate prediction of outcomes following restorative treatment. Omar and Duarte^19^ appraised different programs for digital smile design and concluded that all incorporated facial, dentogingival, and dental esthetic parameters.^19^

In accordance with a previous ITI consensus statement, we recommend that immediate provisional restorations be provided whenever possible following immediate implant placement, to optimize the esthetic outcome.^4^ The use of online planning software greatly facilitates communication between the surgeon, prosthodontist, and dental technician. Through the transfer of digital data between these team members, a provisional implant‐retained restoration with the desired form and shape can be fabricated and placed immediately. The dental technician can create an emergence profile that preserves soft‐tissue volume around the implant.

## 4 CONCLUSION

According to our clinical experience, proper smile design planning facilitates accurate implant placement and provisionalization that supports existing soft‐tissue architecture. This leads to excellent healing and maturation of the peri‐implant tissues in cases with prior root resorption.

## CONFLICT OF INTEREST

None declared.

## AUTHOR CONTRIBUTIONS

KC: performed the surgery and participated in the case study, wrote the original manuscript, and edited and reviewed the final manuscript; GI: performed the prosthetic treatment and was involved in the conception of the study, helped write the original manuscript, and edited and reviewed the final manuscript; SK: participated in the case study and analyzed the data.

## ETHICAL APPROVAL

The patient gave permission to the authors to use the images and to publish the case report.
